# Klinisches und immunpathologisches Spektrum des Immunglobulin‐M‐Pemphigoids: eine multizentrische Fallserie

**DOI:** 10.1111/ddg.15838_g

**Published:** 2025-12-11

**Authors:** Kaan Yilmaz, Stephanie Goletz, Christoph M. Hammers, Katharina Boch, Claudia Zeidler, Henning Wiegmann, Estelle Bergmann, Tina Rastegar Lari, Onur Dikmen, Nina Häring, Robert Strohal, Andreas Kleinheinz, Eva N. Hadaschik, Nina van Beek, Sonja Ständer, Enno Schmidt

**Affiliations:** ^1^ Klinik für Dermatologie Allergologie und Venerologie Universität zu Lübeck Lübeck Deutschland; ^2^ Klinik für Dermatologie Venerologie und Allergologie Medizinische Fakultät Mannheim der Universität Heidelberg Mannheim Deutschland; ^3^ Lübecker Institut für Experimentelle Dermatologie Universität zu Lübeck Lübeck Deutschland; ^4^ Klinik und Poliklinik für Dermatologie Universität Regensburg Regensburg Deutschland; ^5^ Klinik für Dermatologie Venerologie und Allergologie Universität zu Kiel Kiel Deutschland; ^6^ Kompetenzzentrum Chronischer Pruritus Klinik für Hautkrankheiten Universität Münster Münster Deutschland; ^7^ Institut für Pathologie Charité – Universitätsmedizin Berlin Berlin Deutschland; ^8^ Klinik für Dermatologie und Venerologie Landeskrankenhaus Feldkirch Feldkirch Österreich; ^9^ Klinik für Dermatologie Elbe Klinikum Buxtehude Buxtehude Deutschland; ^10^ Klinik für Dermatologie Universität Essen Essen Deutschland

**Keywords:** blasenbildende Autoimmundermatosen, IgM, Pemphigoid, autoimmune bullous diseases, IgM, immunobullous diseases, pemphigoid

## Abstract

**Hintergrund und Zielsetzung:**

Die Pemphigoid‐Erkrankungen werden primär durch IgG‐ oder IgA‐Autoantikörper gegen die kutane Basalmembranzone (BMZ) vermittelt. Obwohl rezente Arbeiten auf die Existenz einer ausschließlich IgM‐vermittelten Pemphigoid‐Erkrankung hindeuten, fehlt bislang eine umfassendere Untersuchung.

**Patienten und Methodik:**

In dieser prospektiven multizentrischen Studie wurden zehn Patienten mit ausschließlich IgM‐Ablagerungen entlang der BMZ in der direkten Immunfluoreszenz (IF) eingeschlossen. Zirkulierende IgM‐Autoantikörper wurden mittels indirekter IF und Immunoblot mit rekombinantem BP180 nachgewiesen.

**Ergebnisse:**

Die Kohorte umfasste vier Frauen und sechs Männer mit einem medianen Alter von 77 Jahren, die überwiegend Prurigo‐artige Hautveränderungen ohne Blasenbildung und Schleimhautbeteiligung aufwiesen. In der indirekten IF auf NaCl‐separierter humaner Spalthaut zeigten die Seren von neun Patienten lineare IgM‐Ablagerungen am Dach der artifiziellen Blase. Bei vier Patienten konnte eine IgM‐Reaktivität gegen BP180 nachgewiesen werden, bei keinem jedoch gegen BP230. Bei keinem der Kontrollpatienten (n*  =  *100) wurden IgM‐Ablagerungen an der BMZ in der direkten IF beobachtet. Eine IgM‐Reaktivität gegen die BMZ im Serum wurde bei 1 von 30 Kontrollpatienten mit chronischem Pruritus beziehungsweise bei 3 von 60 Kontrollpatienten ohne chronischen Pruritus nachgewiesen.

**Schlussfolgerungen:**

Das IgM‐Pemphigoid ist durch gewebsgebundene, exklusive IgM‐Autoantikörper gegen die BMZ, serologische IgM‐Reaktivität mit BP180 als primärem Zielantigen, überwiegend nichtbullösen klinischen Phänotyp, fehlende Schleimhautbeteiligung und eher milden Krankheitsverlauf gekennzeichnet. Unsere Ergebnisse deuten darauf hin, dass das IgM‐Pemphigoid eine eigenständige Entität darstellen könnte.

## EINLEITUNG

Die Pemphigoid‐Erkrankungen (PE) sind eine heterogene Gruppe subepidermal blasenbildender Autoimmundermatosen (BAID), die durch Autoantikörper gegen Strukturproteine der kutanen Basalmembranzone (BMZ) gekennzeichnet sind. Die Diagnosestellung basiert auf drei zentralen Säulen: dem klinischen Bild, der direkten Immunfluoreszenzmikroskopie (IF) einer periläsionalen Probebiopsie sowie serologischen Untersuchungen.^1^, ^2^, ^3^ In der direkten IF zeigen sich bei PE typischerweise lineare Ablagerungen von IgG, Komplementfaktor C3 und/oder IgA entlang der BMZ. Klinisch manifestiert sich die Autoantikörper‐induzierte Beeinträchtigung der dermo‐epidermalen beziehungsweise ‐epithelialen Adhäsion meist in Form prall gespannter Blasen und Erosionen an Haut oder angrenzenden Schleimhäuten.^1^

Basierend auf dem Zielantigen, dem vorherrschenden Autoantikörper‐Isotyp und dem klinischen Befund werden derzeit sechs PE‐Subtypen unterschieden: bullöses Pemphigoid, Schleimhautpemphigoid (SHP), Anti‐p200‐Pemphigoid, Pemphigoid gestationis, lineare IgA‐Dermatose (LAD) sowie die Epidermolysis bullosa acquisita (EBA). Obwohl die Präsenz gewebsgebundener IgM‐Autoantikörper routinemäßig mittels direkter IF untersucht wird, hat das Konzept einer ausschließlich IgM‐vermittelten PE in den vergangenen Jahrzehnten zu wissenschaftlichen Kontroversen geführt.^5^ Bisherige Studien zur pathogenetischen Rolle von IgM bei den PE sind rar und beschränken sich überwiegend auf Einzelfallberichte von IgM‐(bullösem) Pemphigoid,^6^, ^7^, ^8^, ^9^, ^10^ IgM‐SHP,^11^, ^12^, ^13^ und IgM‐EBA.^14^, ^15^, ^16^ In den letzten Jahren wurde das IgM‐Pemphigoid als mögliche eigenständige klinische Entität mit charakteristischen immunpathologischen Merkmalen in den Fokus gerückt.^17^ Das Spektrum klinischer Manifestationen, histopathologischer und immunologischer Befunde sowie therapeutischer Ansprechraten wurde bislang jedoch nicht prospektiv untersucht. In der vorliegenden prospektiven Studie wurden zehn PE‐Patienten mit exklusiver Anti‐BMZ‐IgM‐Reaktivität in der direkten IF charakterisiert.

## PATIENTEN, MATERIAL UND METHODIK

### Patienten

Diese prospektive multizentrische Fallstudie umfasste zehn Patienten aus zwei dermatologischen Zentren der Primärversorgung in Lübeck und Hannoversch Münden, Deutschland, sowie aus fünf dermatologischen Zentren der Sekundär‐ beziehungsweise Tertiärversorgung in Deutschland (Lübeck, Kiel, Buxtehude, Essen) und Feldkirch, Österreich. Zu den Einschlusskriterien zählten ein klinischer Verdacht auf eine BAID, der zur Durchführung einer direkten IF‐Mikroskopie an einer periläsionalen Hautbiopsie führte, sowie der Nachweis linearer Anti‐BMZ‐Autoantikörper ausschließlich vom IgM‐Isotyp mittels direkter IF. Die Diagnosestellung erfolgte im Rahmen der Routinediagnostik im Autoimmunlabor der Universität zu Lübeck im Zeitraum von Oktober 2020 bis März 2022.

Für serologische Untersuchungen wurden zwei Kontrollgruppen herangezogen: *(1)* 60 Patienten von der Universität zu Lübeck mit klinischem Verdacht auf eine BAID, bei denen diese jedoch mittels immunpathologischer Verfahren ausgeschlossen wurde, sowie *(2)* 30 Patienten von der Universität Münster mit juckenden Dermatosen außer BAID. Alle Kontrollpersonen waren über 70 Jahre alt. Die Patienten der zweiten Kontrollgruppe wurden hinsichtlich der zugrunde liegenden Krankheitsentität, der ätiologischen Klassifikation gemäß dem *International Forum for the Study of Itch* (IFSI)^18^ sowie der Pruritusintensität in den letzten 24 Stunden mittels *Worst Itch Numeric Rating Scale* (WI‐NRS) und *Average Itch Numeric Rating Scale* (AI‐NRS) erfasst, wie zuvor beschrieben.^19^, ^20^

Eine dritte Kontrollgruppe umfasste 100 konsekutive Patienten über 70 Jahre, bei denen im Rahmen des Verdachts auf eine BAID eine direkte IF einer periläsionalen Probebiopsie aus lichtexponierter Haut (Arm, Bein oder Stamm) durchgeführt wurde, jedoch keine linearen Ablagerungen von IgG, IgA und/oder C3 an der kutanen BMZ nachgewiesen werden konnten.

### Immunfluoreszenz‐Untersuchungen

Die direkte IF‐Mikroskopie periläsionaler Hautbiopsien erfolgte gemäß etablierten Standardprotokollen. Die Proben wurden mit Fluoresceinisothiocyanat (FITC)‐konjugierten Antikörpern gegen humanes IgG (Dako, Kalifornien, USA), IgA (Euroimmun, Lübeck, Deutschland), IgM (Euroimmun, Lübeck, Deutschland) sowie C3 (MP Biomedicals, Kalifornien, USA) angefärbt.^21^

Die indirekte IF‐Mikroskopie auf 1 M NaCl‐separierter humaner Spalthaut zum Nachweis zirkulierender IgG‐, IgA‐ und IgM‐Autoantikörper (jeweils 1:2 verdünnt) wurde wie zuvor beschrieben durchgeführt.^17^ Des Weiteren erfolgte eine indirekte IF mit einem BIOCHIP^®^‐Mosaik (Euroimmun, Lübeck, Deutschland), bestehend aus 1 M NaCl‐gespaltener Primatenhaut, rekombinantem tetrameren BP180‐NC16A sowie HEK293‐Zellen, die mit der C‐terminalen globulären Domäne von BP230 transfiziert waren. Hierbei kamen FITC‐markierte Anti‐IgM‐Antikörper (MP Biomedicals, Kalifornien, USA; 1:50 und 1:100 verdünnt) zur Anwendung. Die Seren wurden in einer Verdünnung von 1:10 getestet. Die Auswertung aller IF‐Proben erfolgte durch in diesem Bereich speziell geschulte und erfahrene Ärzte.

### Immunoblot‐Untersuchungen

Die Immunoblot‐Analysen wurden mit folgenden rekombinanten BP180‐Fragmenten durchgeführt: *(1)* BP180 NC16A (aa490–562), *(2)* rekombinante BP180‐Ektodomäne (BP180ec; aa490–1.497) und *(3)* einem rekombinanten C‐terminalen Fragment von BP180 (BP180(ec)3; aa1.024–1.270), gemäß zuvor publizierten Protokollen.^17^

### Histopathologie

Läsionale Hautbiopsate wurden im Rahmen der Routinediagnostik entnommen und nach Standardverfahren mit Hämatoxylin‐Eosin (HE) gefärbt und ausgewertet.

### Ethikvotum

Diese Studie entspricht den Grundsätzen der Deklaration von Helsinki und wurde von den Ethikkommissionen der Universität zu Lübeck (22‐129, 23–535) und der Universität Münster (2023‐487‐b‐S) genehmigt. Alle in diesem Manuskript beschriebenen Patienten haben ihre schriftliche Einwilligung zur Veröffentlichung ihrer Falldetails erteilt.

## ERGEBNISSE

Die Studie umfasste vier weibliche und sechs männliche Patienten mit einem medianen Alter von 77 Jahren (Bereich: 60–98 Jahre) zum Zeitpunkt der Diagnosestellung des IgM‐Pemphigoids. Das mediane diagnostische Intervall, also der Zeitraum zwischen von Patienten angegebenem Symptombeginn und abschließender Diagnosestellung, betrug 12 Monate und variierte zwischen 2 und 26 Monaten (Tabelle 1).

**TABELLE 1:** Demographische, klinische und histopathologische Merkmale der Patienten mit IgM‐Pemphigoid (n=10).

**Alter bei Diagnose; Jahre**	
Mittelwert (SD)	78,4 (11,4)
Median (Spanne)	77 (60–98)
**Geschlecht**	**n**
Weiblich	4
Männlich	6
**Zeitspanne vom Symptombeginn bis zur Diagnose; Monate**	
Mittelwert (SD)	13 (8,4)
Median (Spanne)	12 (2–26)
**Verdachtsdiagnosen bei Erstvorstellung**	**n**
Chronische Prurigo	5
Arzneimittelexanthem	2
Ekzem	1
Parapsoriasis	1
Bullöses Pemphigoid	1
**Klinische Merkmale**	
Juckreiz	10
Erythem/urtikarielle Läsionen	9
Exkoriierte Papeln/Plaques	7
Lichenifikation	6
Erosionen	3
Blasen	2*
Hyperpigmentierung	1
Schleimhautbeteiligung	0
**Histologische Merkmale**	
Lymphozytäre Infiltrate	9
Eosinophile Granulozyten	6
Parakeratose	5
Spongiose	5
Akanthose	5
Neutrophile Granulozyten	3
Dermales Ödem	3
Hypergranulose	2
Hypogranulose	1
Subepidermale Spaltbildung	1/

*Abk*.: n, Anzahl; SD, Standardabweichung

* Anamnestische Angaben; es wurden keine Blasen bei Erstvorstellung oder Verlaufskontrollen festgestellt.

### Klinisches Bild

**ABBILDUNG 1:**
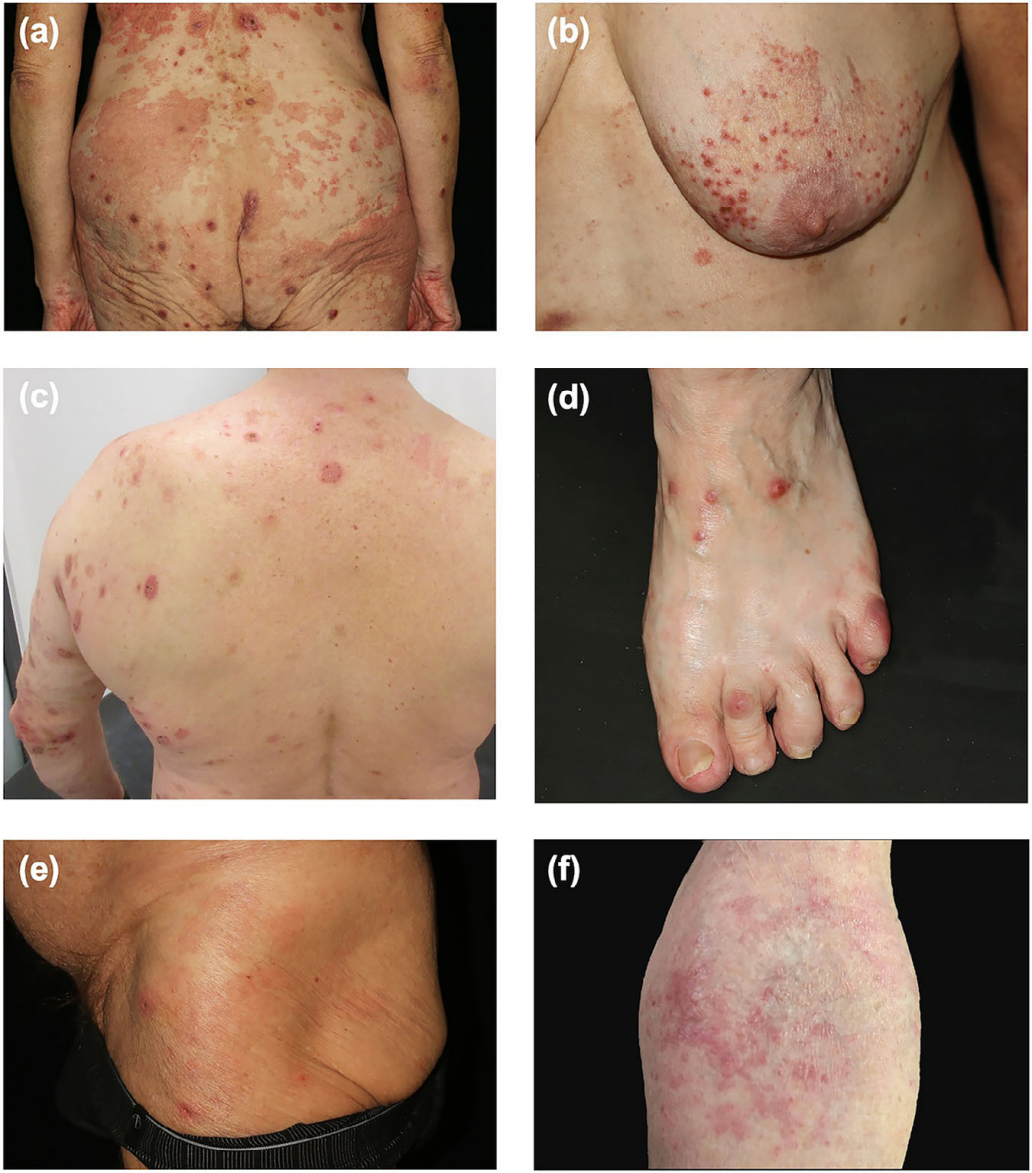
Klinische Manifestationen des IgM‐Pemphigoids. (a) Konfluierende, erythematöse, scharf begrenzte urtikarielle Plaques sowie multiple exkoriierte Papeln an Gesäß und unterem Rücken der Patientin 6. (b) Mehrere gruppiert stehende, stecknadelkopfgroße, exkoriierte Papeln auf erythematösem Grund an der Brust der Patientin 6. (c) Disseminierte, scheibenförmige erythematöse Plaques am oberen Rücken und linken Arm bei Patient 5. (d) Erythematöse Papeln und Plaques auf dem Fußrücken links der Patientin 6. (e) Erythematöse Makulae und Plaques mit wenigen exkoriierten Papeln an der linken Hüfte bei Patient 8. (f) Erythematöse Makulae in der Ellenbeuge der Patientin 10.

Alle Patienten wiesen eine von Pruritus begleitete Hauterkrankung auf. Erytheme und urtikarielle Läsionen waren mit einer Prävalenz von 90 % die häufigsten Manifestationen, gefolgt von exkoriierten Papeln und Plaques (bei 70 %) sowie Lichenifikation (bei 60 %). Erosionen wurden bei drei Patienten (30 %) beobachtet, während ein Patient (10 %) Hyperpigmentierungen aufwies. Zwei Patienten (20 %) berichteten anamnestisch über Blasen, wobei diese weder bei der Erstvorstellung noch bei den Verlaufskontrollen klinisch objektiviert werden konnten. Bei keinem der Patienten konnten orale, nasale, okuläre oder anogenitale Schleimhautläsionen festgestellt werden (Tabelle 1, Abbildung 1).

Bei allen Patienten erfolgte eine direkte IF‐Mikroskopie zum Ausschluss einer PE. Die klinischen Verdachtsdiagnosen umfassten chronische Prurigo bei fünf Patienten, Arzneimittelexanthem im Zusammenhang mit Hydrochlorothiazid beziehungsweise Lamotrigin bei zwei Patienten sowie Ekzem, Parapsoriasis und bullöses Pemphigoid bei jeweils einem Fall (Tabelle 1, Tabelle S1).

### Histopathologische Befunde

**ABBILDUNG 2:**
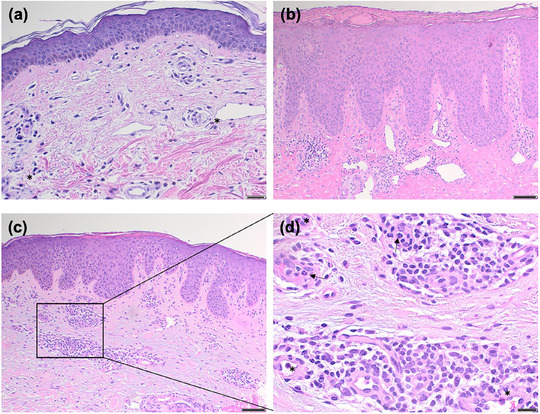
Histopathologisches Spektrum des IgM‐Pemphigoids. (a) Oberflächlich perivaskuläre Dermatitis mit leichtem dermalem Ödem und milden lymphohistiozytären Infiltraten unter Beimischung vereinzelter eosinophiler Granulozyten (Sternchen) bei Patientin 3 (Hämatoxylin‐Eosin‐Färbung [HE], Originalvergrößerung ×  10; Maßstabsbalken: 100 µm). (b) Chronische, noduläre, Prurigo‐ähnliche Dermatose mit Parakeratose, unregelmäßiger Akanthose und Hypergranulose bei Patientin 6. In der oberen Dermis zeigen sich Fibrose und herdförmige lymphohistiozytäre Infiltrate (HE, ×  10; Maßstabsbalken: 100 µm). (c) Milde spongiotische und psoriasiforme Dermatitis bei Patientin 10 (HE, ×  10; Maßstabsbalken: 100 µm). (d) Perivaskuläre lymphozytäre Infiltrate mit beigemischten neutrophilen Granulozyten (Pfeile) und wenigen eosinophilen Granulozyten (Sternchen) in höherer Vergrößerung von (c) (HE, ×  40; Maßstabsbalken: 20 µm).

Epidermale Veränderungen zeigten sich häufig in den läsionalen Hautbiopsaten, darunter Parakeratose (5 Patienten), Akanthose (5 Patienten), Spongiose (5 Patienten), Hypergranulose (2 Patienten) sowie Hypogranulose (1 Patientin). Eine subepidermale Spaltbildung wurde in einem Fall nachgewiesen. Ödem der oberen Dermis, charakteristisch für urtikarielle Dermatitis, zeigte sich in drei Fällen. Die dermalen Infiltrate bestanden überwiegend aus Lymphozyten (9 Patienten), teils mit beigemischten eosinophilen Granulozyten (6 Fälle) und neutrophilen Granulozyten (3 Fälle) (Tabelle 1, Abbildung 2).

### Immunpathologische Merkmale

**ABBILDUNG 3:**
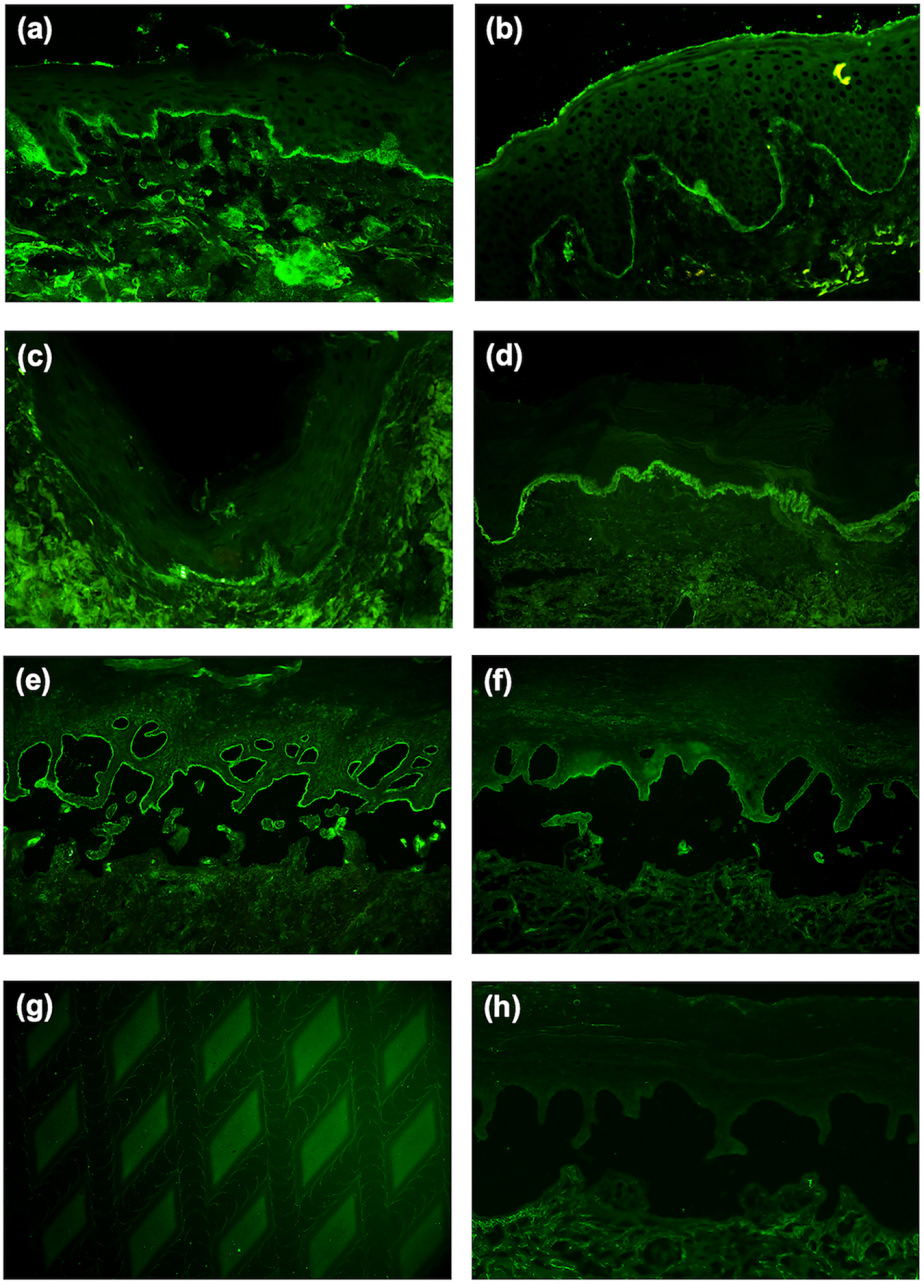
Immunpathologische Merkmale des IgM‐Pemphigoids. (a‐c) Direkte Immunfluoreszenz (IF)‐Mikroskopie einer periläsionalen Hautbiopsie mit linearen IgM‐Ablagerungen an der kutanen Basalmembranzone (BMZ) bei (a) Patient 9, (b) Patientin 10 und (c) Patientin 3. (d) Dicke, homogene, bandförmige IgM‐Ablagerungen an der BMZ bei einem Patienten mit Lupus erythematodes zum Vergleich. (e) Indirekte IF‐Mikroskopie an humaner, mit 1 M NaCl gespaltener Haut mit IgM‐Ablagerungen auf der epidermalen Seite der artifiziellen Blase bei Patientin 6. (f) Lineare IgM‐Ablagerungen am Blasendach NaCl‐gespaltener Haut mittels multiplexer IF‐BIOCHIP^®^‐Mosaiktechnik (Euroimmun, Lübeck, Deutschland) bei Patient 4. (g) IgM‐Autoantikörper gegen BP180 NC16A im BIOCHIP^®^‐Mosaik mit rekombinantem BP180 bei Patientin 3. (h) Keine IgM‐Ablagerungen bei indirekter IF‐Mikroskopie an NaCl‐gespaltener humaner Haut bei einem Kontrollpatienten mit atopischer Dermatitis.

Die direkte IF‐Mikroskopie aller periläsionalen Hautbiopsien zeigte ausschließlich lineare IgM‐Ablagerungen entlang der BMZ ohne Nachweis von IgG‐, IgA‐ oder C3‐Ablagerungen (Abbildung 3a–c). Hierbei konnte in neun Fällen nicht zwischen einem n‐Muster oder u‐Muster unterschieden werden, während ein Biopsat ein n‐Muster zeigte (Tabellen 2, S2). In 100 konsekutiv entnommenen Gewebeproben sonnenexponierter Hautareale von Patienten über 70 Jahren mit klinischem Verdacht auf eine BAID, jedoch ohne IgG‐, IgA‐ oder C3‐Ablagerungen an der BMZ, fanden sich in keinem Fall lineare IgM‐Ablagerungen. In dieser Kontrollgruppe wurden bei 3 % der Fälle *cytoid bodies* und bei 1 % granuläre IgM‐Ablagerungen nachgewiesen (Tabelle S3).

**TABELLE 2:** Immunpathologische Merkmale von Patienten mit IgM‐Pemphigoid (n=10)

	n
**Lineare Ablagerungen von IgM an der kutanen BMZ bei direkter IF**	
n‐Muster	1
u‐Muster	0
Kein Muster	9
Zusätzliche IgG‐/IgA‐Reaktivität	0
**Indirekte IF auf NaCl‐separierter humaner Spalthaut**	
IgM am Blasendach	9
IgM am Blasenboden	0
Negativ	1
Zusätzliche IgG‐/IgA‐Reaktivität	0
**Zielantigen**	
BP180 NC16A (IgM)	3
BP180‐Ektodomäne (IgM)	2
BP180(ec)3 (C‐terminal, IgM)	0
BP230 (IgM)	0
Unbekannt	6
BP180 NC16A (IgG)	1^*^

*Abk*.: n, Anzahl; BMZ, Basalmembranzone; IF, Immunfluoreszenz; ec, Ektodomäne

* Zusätzliche IgG‐Reaktivität gegen BP180 NC16A durch ELISA (27 U/ml; Normwert, < 20 U/ml) bei Patientin 3.

**ABBILDUNG 4:**
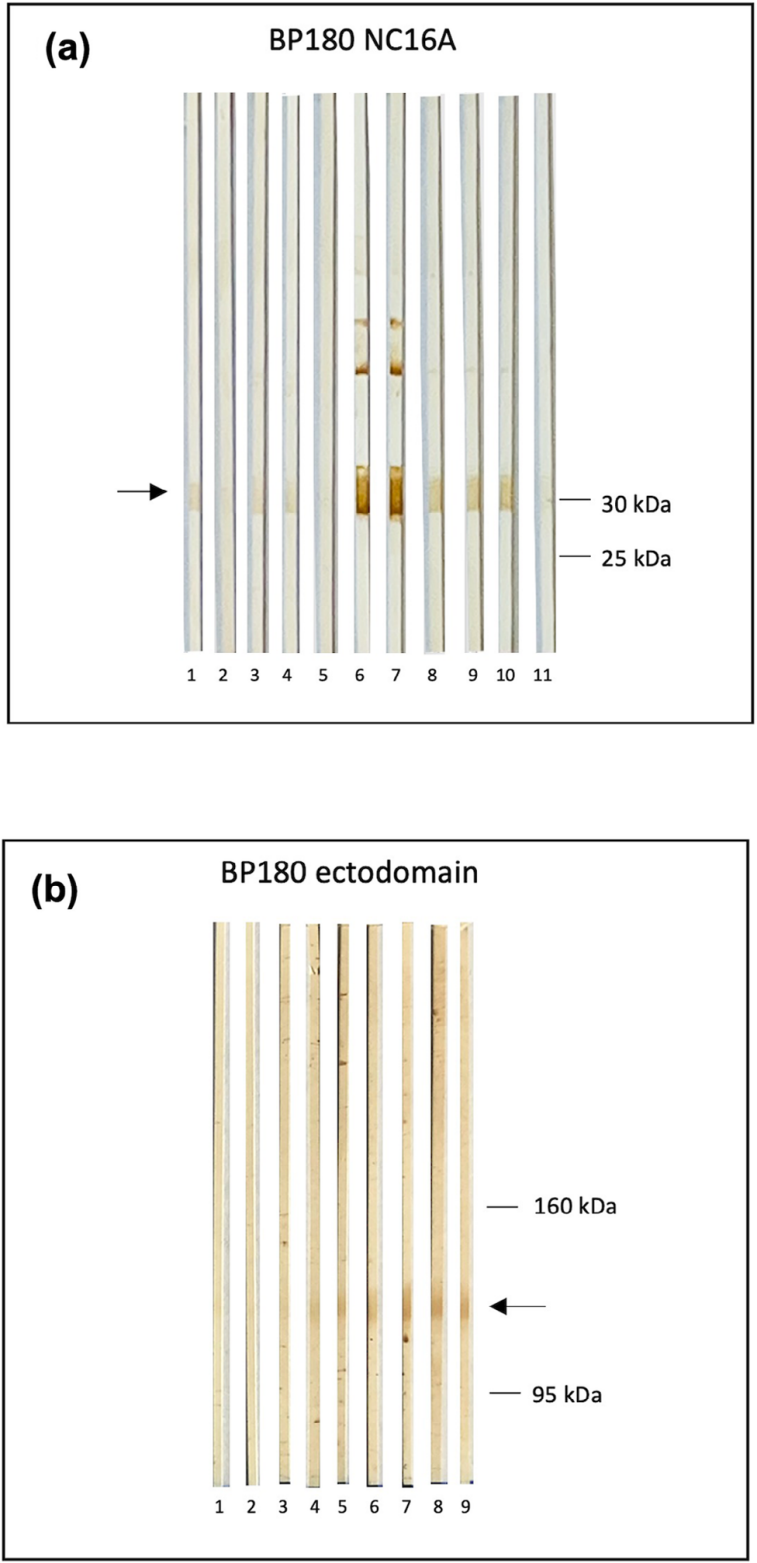
Immunoblot‐Analysen beim IgM‐Pemphigoid. (a) Immunoblot‐Analysen mit rekombinantem BP180‐NC16A. Eine IgM‐Serumreaktivität zeigte sich bei Patientin 3 (Spuren 9 und 10), nicht jedoch bei Patient 4 (Spur 11). Sera gesunder Blutspender dienten als Negativkontrollen (Spuren 1–5). Sera von drei zuvor beschriebenen Patienten mit IgM‐Pemphigoid^17^ wurden als Positivkontrollen verwendet (Spuren 6–8). (b) Bei Immunoblotting mit der rekombinanten BP180‐Ektodomäne zeigten Patientin 6 und Patient 7 eine IgM‐Serumreaktivität (Spuren 8 bzw. 9). Normale Humansera sind in den Spuren 1–5 dargestellt, zwei zuvor beschriebene Patienten mit IgM‐Pemphigoid^17^ in den Spuren 6 und 7. Die Migrationspositionen der rekombinanten Proteine sind durch Pfeile markiert, die molekularen Gewichtsmarker in kDa sind rechts dargestellt

Bei neun von zehn Patienten mit IgM‐Pemphigoid konnten zirkulierende IgM‐Autoantikörper mittels indirekter IF‐Mikroskopie auf NaCl‐separierter humaner Spalthaut detektiert werden. In allen Fällen banden IgM‐Ablagerungen an die epidermale Seite der artifiziellen Blase (Abbildung 3e). Die Immunoblot‐Untersuchungen mit der rekombinanten NC16A‐Domäne von BP180 (Abbildung 4a) sowie der rekombinanten BP180‐Ektodomäne (Abbildung 4b) zeigten IgM‐Reaktivität bei drei beziehungsweise zwei Patienten. Eine Patientin (Patientin 6, Tabelle S2) wies in beiden Immunoblot‐Verfahren IgM‐Reaktivität auf. Ein weiterer Immunoblot mit einem C‐terminalen Fragment von BP180 (BP180(ec)3) ergab bei keinem Patienten eine IgM‐Reaktivität. Zirkulierende IgG‐ oder IgA‐Autoantikörper wurden bei keinem der Patienten durch indirekte IF nachgewiesen. Lediglich eine Patientin (Patientin 3) zeigte eine zusätzliche geringe IgG‐Reaktivität gegen BP180 NC16A im ELISA (27 U/ml; Normwert < 20 U/ml) (Tabelle 2).

Mittels BIOCHIP^®^‐Mosaik wurden keine IgM‐Autoantikörper gegen BP230 detektiert. Hingegen konnte die IgM‐Reaktivität gegen die epidermale Seite der NaCl‐separierten humanen Spalthaut bei sieben von neun untersuchten Seren bestätigt werden, wobei nur eines der drei Immunoblot‐positiven Seren eine IgM‐Reaktivität gegen BP180 NC16A zeigte (Tabelle S2, Abbildung 3f–g). In vier Fällen standen Serumproben zu verschiedenen Zeitpunkten des Krankheitsverlaufs zur Verfügung; in keinem Fall wurde ein Isotypwechsel beobachtet (Daten nicht dargestellt).

In den serologischen Kontrollgruppen wurden zirkulierende IgM‐Autoantikörper bei einem von 30 Patienten (3,33 %) mit gut charakterisierten, pruritischen Dermatosen außer PE sowie bei drei von 60 Patienten (5 %) mit klinischem Verdacht, jedoch immunopathologisch ausgeschlossener PE nachgewiesen (Abbildung 3h; Tabellen S4, S5).

### Therapieansprechen

Der Krankheitsverlauf wurde bei acht Patienten über einen medianen Zeitraum von 10 Monaten (Spanne: 4–28 Monate) dokumentiert. Zwei Patienten konnten im Verlauf der Nachbeobachtung nicht weiterverfolgt werden. Der krankheitsspezifische Therapieerfolg wurde nach den Kriterien eines internationalen Konsensus erfasst.^1^ (Tabelle S1).

Alle Patienten wurden topisch mit potenten oder hochpotenten Glukokortikosteroiden behandelt. Vier Patienten wurden zusätzlich systemisch, unter anderem mit Prednisolon, Azathioprin, Mycophenolatmofetil (MMF), Doxycyclin, Dapson oder Rituximab therapiert. Von den fünf ausschließlich topisch behandelten Patienten erreichten drei eine komplette Remission bei Therapiefreiheit, während zwei Patienten eine partielle Remission unter minimaler Erhaltungstherapie erzielten.

In der systemisch behandelten Gruppe erreichten zwei Patienten eine partielle Remission unter Monotherapie mit Dapson beziehungsweise Azathioprin. Bei einem weiteren Patienten kam es trotz sequenzieller Behandlung mit Doxycyclin, Azathioprin und Mycophenolatmofetil (MMF) in Kombination mit oralem Prednisolon zum Rezidiv. Ausreichende Krankheitskontrolle konnte erst durch die zusätzliche Gabe von Rituximab erzielt werden (Patient 5, Tabelle S1).

## DISKUSSION

Die IgM‐vermittelte PE wurde erst in jüngster Zeit als potenziell eigenständige Entität vorgeschlagen. Erste Untersuchungen beschrieben zwar die klinische Heterogenität der betroffenen Patienten, stellten jedoch die pathogenetische Relevanz von IgM‐Autoantikörpern gegen die kutane BMZ infrage.^5^ Infolgedessen wurde kein spezifisches Zielantigen des IgM‐Pemphigoids identifiziert, und die beobachteten IgM‐Ablagerungen wurden als Ausdruck einer Überempfindlichkeitsreaktion gedeutet, wie sie typischerweise bei Urtikaria, leukozytoklastischer Vaskulitis oder allergischer Kontaktdermatitis vorkommen.^7^ Interessanterweise wurde in einer frühen Studie von Velthuis et al. lediglich bei einem von 25 untersuchten Fällen tatsächlich von einem bullösen Pemphigoid ausgegangen – mit kutanen Blasen, IgM‐ und C3‐Ablagerungen entlang der BMZ sowie zirkulierenden Anti‐BMZ‐Autoantikörpern.^5^ Abgesehen von diesem Einzelfall gelang es früheren Arbeiten nicht, IgM‐Autoantikörper im Serum hinreichend zu charakterisieren.^24^ Im weiteren Verlauf der wissenschaftlichen Auseinandersetzung wurde der Begriff „lineare IgM‐Dermatose der Schwangerschaft“ eingeführt, um ein klinisches Bild mit vorübergehenden juckenden, erythematösen Papeln und urtikariellen Läsionen – typischerweise im dritten Trimenon – zu beschreiben, das mit linearen IgM‐Ablagerungen an der kutanen BMZ einhergeht.^25^, ^26^ Obwohl dieses Konzept einer neuartigen schwangerschaftsassoziierten Entität bislang keine breite Akzeptanz gefunden hat, wurden zirkulierende IgM‐Autoantikörper gegen BP180 und BP230 bei 10–14 % der schwangeren Frauen nachgewiesen, was auf eine mögliche Assoziation zwischen Schwangerschaft und geringer kutaner IgM‐Autoreaktivität hinweist.^27^ In unserer Kohorte befand sich keine der Patientinnen im reproduktiven Alter, und die Altersverteilung entsprach jener des bullösen Pemphigoids, das typischerweise in einem höheren Lebensalter (spätes 7. Lebensjahrzehnt) manifest wird.^1^ Bemerkenswerterweise wurden zwischen Oktober 2020 und März 2022 im Autoimmunlabor der Klinik für Dermatologie, Allergologie und Venerologie der Universität zu Lübeck insgesamt 527 Patienten mit PE diagnostiziert, darunter lediglich zehn Fälle mit IgM‐Pemphigoid – entsprechend etwa 1,9 % aller PE‐Fälle.

Einige Fälle von PE mit IgM‐Reaktivität gegen die kutane BMZ wurden in der Literatur mit einer Waldenström‐Makroglobulinämie assoziiert.^28^, ^29^ In unserer Fallserie ließ sich diese hingegen bei keinem der Patienten nachweisen. Trotz zahlreicher Bemühungen blieb das Zielantigen der IgM‐Autoantikörper bei PE bislang weitestgehend unklar – mit Ausnahme eines Einzelfalls einer IgM‐vermittelten EBA mit Nachweis von IgM‐Autoantikörpern gegen Typ‐VII‐Kollagen.^14^ Erst eine jüngere Studie konnte zirkulierende IgM‐Autoantikörper gegen BP180 bei allen drei untersuchten Patienten mit IgM‐Pemphigoid nachweisen.^17^ Im Einklang damit konnte bei einem Patienten mit IgM‐Pemphigoid mittels hochauflösender Mikroskopie BP180 als Autoantigen identifiziert werden, wobei insbesondere das C‐terminale Ende von BP180 als potenzielles Zielantigen vermutet wurde.^9^

In der vorliegenden Studie konnten bei neun von zehn Patienten zirkulierende IgM‐Autoantikörper gegen die BMZ detektiert werden, während vier von zehn Patienten IgM‐Autoantikörper gegen BP180 aufwiesen. Am häufigsten richtete sich die Autoantikörperreaktivität gegen den extrazellulären Abschnitt der 16. nicht‐kollagenen Domäne (NC16A) (in 30 %), die zugleich die immundominante Region von BP180 beim bullösen Pemphigoid darstellt, gefolgt von der BP180‐Ektodomäne. Bei den übrigen sechs Patienten konnte kein spezifisches Zielantigen identifiziert werden, während in all diesen Fällen IgM‐Ablagerungen an der epidermalen Seite der artifiziellen Blase bei der indirekten IF‐Mikroskopie beobachtet wurden. Dieses Ergebnis lässt sich möglicherweise sowohl durch niedrigere Konzentrationen von IgM‐Autoantikörpern in diesen Fällen als auch durch die Tatsache erklären, dass die eingesetzten Immunoblot‐ und multivariaten IF‐Methoden nicht für den Nachweis von IgM‐Reaktivität optimiert waren. Alternativ könnten die IgM‐Autoantikörper gegen andere epidermale Komponenten der BMZ wie BP230, α6β4‐Integrin oder Plektin gerichtet sein, wobei deren Identifikation durch das Fehlen validierter Nachweismethoden zur Erfassung IgM‐spezifischer Autoreaktivität gegen diese Zielantigene erschwert wird. Interessanterweise ließ sich ein Bindungsmuster der linearen IgM‐Ablagerungen bei der direkten IF‐Mikroskopie lediglich bei einem Patienten nachweisen. Dies steht im Kontrast zu anderen Varianten der PE, bei denen ein n‐ oder u‐Muster bei circa 75 % der Fälle beobachtet wird.^30^ Diese Diskrepanz könnte auf strukturelle Eigenschaften von IgM zurückgeführt werden, welches überwiegend als Pentamer vorliegt.^31^ Diese molekulare Konfiguration könnte die Ausbildung oder Detektion eines klaren Bindungsmusters bei der direkten IF‐Mikroskopie beeinträchtigen.

Zur weiteren Aufklärung der bestehenden Kontroverse über die diagnostische Spezifität linearer IgM‐Ablagerungen wurden in die vorliegende Studie mehrere Kontrollgruppen eingeschlossen. In Biopsaten lichtexponierter Haut von 100 Kontrollpersonen ohne BAID ließen sich mittels direkter IF‐Mikroskopie keine linearen IgM‐Ablagerungen entlang der BMZ nachweisen. Nebenbefundlich wurden bei 3 % der Fälle *cytoid bodies* und bei 1 % granuläre IgM‐Ablagerungen identifiziert (Tabelle S3). IgM‐positive *cytoid bodies*, insbesondere bei gehäuftem und aggregiertem Auftreten, sind charakteristisch für Erkrankungen wie Lupus erythematodes und Lichen planus.^3^ ^2^ Vereinzelte *cytoid bodies* können hingegen in einer Vielzahl chronisch‐entzündlicher Dermatosen auftreten, darunter kutane *Graft‐versus‐Host*‐Erkrankung, Lupus erythematodes, Erythema multiforme, leukozytoklastische Vaskulitis, Elastosis perforans serpiginosa sowie in lichtexponierter Haut gesunder Individuen.^33^, ^34^, ^35^ Eine umfassende Analyse von über 1000 Hautproben mittels direkter IF‐Mikroskopie ergab die Präsenz von *cytoid bodies* bei 10,8 % der Fälle, wobei bei 72,6 % dieser Fälle der IgM‐Isotyp zu beobachten war.^33^ Die gleiche Studie wies zudem nach, dass granuläre IgM‐Ablagerungen ebenfalls bei chronisch‐entzündlichen Dermatosen wie Lupus erythematodes vorkommen können.^33^

In einem zweiten Schritt wurden immunserologische Untersuchungen mit Seren von Patienten mit gut charakterisierten, chronisch‐juckenden Dermatosen sowie von Patienten mit klinisch vermuteter, aber immunpathologisch ausgeschlossener PE durchgeführt. Entsprechend wurden IgM‐Ablagerungen an der epidermalen Seite der NaCl‐separierten humanen Spalthaut in der indirekten IF‐Mikroskopie nur in Einzelfällen beobachtet (1/30 beziehungsweise 3/60). Bei 6,3 % der Seren konnten mittels Immunoblot IgM‐Antikörper gegen BP180 NC16A nachgewiesen werden (Tabellen S4, S5), was möglicherweise auf die fehlende Optimierung dieser Assays für die Detektion von IgM‐Reaktivitäten zurückzuführen ist. Zusammenfassend legen die Ergebnisse unserer Studie nahe, dass die Diagnosestellung eines IgM‐Pemphigoids auf Grundlage des klinischen Befunds, direkter IF sowie indirekter IF auf NaCl‐separierter humaner Spalthaut erfolgen kann.

Bei der direkten IF‐Mikroskopie ist es essenziell, die für PE typische und diagnostische, lineare Anti‐BMZ‐Reaktivität von bandförmigen IgM‐Ablagerungen, wie sie bei Lupus erythematodes und potenziell auch bei anderen entzündlichen Dermatosen oder lichtexponierter Haut vorkommen können, differenzialdiagnostisch abzugrenzen. In Analogie zu anderen Formen der PE ist das IgM‐Pemphigoid durch scharf begrenzte, dünne und kontinuierliche lineare IgM‐Ablagerungen entlang der BMZ gekennzeichnet (Abbildung 3a–c). Hingegen präsentiert sich der Lupus erythematodes in der Regel mit breiten, bandförmigen, homogenen oder granulären IgM‐Ablagerungen (Abbildung 3d) – ein Muster, das auf Immunkomplexablagerungen beziehungsweise bessere Zugänglichkeit oder Modifikation kutaner Antigene durch die UV‐induzierte Keratinozytenschädigung zurückgeführt wird.^36^, ^37^ Morphologisch ähnliche IgM‐Ablagerungen können jedoch auch in lichtexponierter Haut, anderen chronisch‐entzündlichen Dermatosen sowie bei gesunden Individuen nachgewiesen werden. Dieses Muster ist typischerweise ebenfalls granulär oder bandförmig, jedoch weniger intensiv, oft fokal oder unterbrochen und wird allgemein als unspezifisch eingestuft.^36^, ^38^

Die fehlende Schleimhautbeteiligung bei unseren Patienten steht im Einklang mit früheren Berichten über IgM‐PE, die ausschließlich auf die Haut beschränkt waren^9^, ^39^ oder nur geringfügige orale Läsionen zeigten.^6^ Auch alle bislang beschriebenen Fälle einer IgM‐vermittelten EBA zeigten keine mukosale Beteiligung.^14^, ^15^, ^16^ Demgegenüber sind Schleimhäute in 10–20 % der Fälle beim bullösen Pemphigoid^40^ und bei etwa der Hälfte der Patienten mit EBA betroffen.^41^ Nur wenige Fälle eines IgM‐vermittelten SHP wurden bislang dokumentiert, wobei das Spektrum von milden konjunktivalen Läsionen bis hin zu schwer vernarbender Augenbeteiligung reicht.^11^, ^12^, ^13^

Rezente Studien zeigen, dass zirkulierende Autoantikörper beim IgM‐Pemphigoid BP180 *ex vivo* internalisieren können, ohne jedoch das Komplementsystem zu aktivieren oder eine Blasenbildung zu induzieren.^17^ Diese Befunde decken sich mit unseren Beobachtungen, wonach bei keinem der Patienten C3‐Ablagerungen an der BMZ mittels direkter IF nachweisbar waren. Trotz der bekannten komplementaktivierenden Eigenschaften von IgM wird das Komplementsystem nur bei Bindung an Oberflächenantigene effektiv aktiviert.^42^ Darüber hinaus macht die hexamere Form des IgM – welche wesentlich effizienter in der Komplementaktivierung ist als die pentamere – weniger als 5 % des Gesamt‐IgM aus.^31^, ^43^ Daraus lässt sich ableiten, dass niedrigaffine Interaktionen zwischen überwiegend pentamerem IgM und BP180 zu unzureichender Komplementaktivierung führen könnten, was wiederum mit verminderter Entzündungsreaktion in der Haut und fehlender klinischer und histologischer Blasenbildung einhergehen würde. Frühere Fallberichte mit bullösem Phänotyp eines IgM‐Pemphigoids zeigten regelmäßig lineare C3‐Ablagerungen in Kombination mit IgM‐Autoantikörpern an der BMZ,^6^ wobei die Anwesenheit von gewebsgebundenem C3c nicht zwingend mit klinischer Blasenbildung einherging.^9^, ^17^ Daher erscheint es naheliegend, dass das IgM‐Pemphigoid unter zusätzlicher Beteiligung von Komplement C3 sowie IgG‐ und/oder IgA‐Autoantikörpern aggraviert werden oder einen phänotypischen Wandel erfahren könnte. In zukünftigen Arbeiten muss daher untersucht werden, ob die Diagnose eines IgM‐Pemphigoids, wie in der vorliegenden Studie, zwingend das Fehlen von IgG‐, IgA‐ und C3‐Reaktivität in der direkten IF‐Mikroskopie voraussetzt oder, analog zur LAD, auch Fälle mit schwächerer Ko‐Reaktivität miteinschließt. In diesem Zusammenhang wäre es empfehlenswert, während des Krankheitsverlaufs die direkte IF‐Mikroskopie einer periläsionalen Hautbiopsie zu wiederholen, insbesondere bei Auftreten neuer Hautläsionen oder Veränderungen des klinischen Erscheinungsbildes.

Die Mehrheit unserer Patienten präsentierte sich mit juckenden Papeln und Plaques, Erythemen sowie Lichenifikation, ohne dass Blasen auftraten, was die aktuellen Erkenntnisse zur klinischen Manifestation des IgM‐Pemphigoids untermauert.^17^ Lediglich zwei Patienten berichteten über Blasenbildung vor der Erstvorstellung, die jedoch weder zum Zeitpunkt der initialen Untersuchung noch bei den klinischen Verlaufskontrollen objektiviert werden konnte. In dieser Hinsicht scheint sich das IgM‐Pemphigoid vom klassischen bullösen Pemphigoid zu unterscheiden, obwohl bei letzterem etwa 20 % der Patienten mit nichtbullösen Varianten vorstellig werden, die klinisch einer chronischen Prurigo, einem Ekzem, einer Intertrigo, einem Ecthyma gangraenosum, einer Erythrodermie oder einer toxischen epidermalen Nekrolyse ähneln.^44^

Hervorzuheben ist, dass die durchschnittliche diagnostische Latenz in unserer Kohorte mit 12 Monaten nahezu doppelt so lang war wie beim bullösen Pemphigoid (6,1 Monate).^44^ Diese Verzögerung könnte auf das eher unspezifische und milde klinische Erscheinungsbild des IgM‐Pemphigoids zurückzuführen sein, welches sich zudem in einem insgesamt milden Krankheitsverlauf widerspiegelt: Die Hälfte der Patienten erreichte unter alleiniger Behandlung mit topischen Kortikosteroiden entweder eine vollständige Remission nach Absetzen der Therapie oder eine partielle Remission unter minimaler Therapie innerhalb weniger Monate. Diese Beobachtungen bestätigen aktuelle Berichte, die einen milden Verlauf des IgM‐Pemphigoids nahelegen.^17^

Zusammenfassend charakterisiert die vorliegende Arbeit das IgM‐Pemphigoid als eine eigenständige und potenziell unterdiagnostizierte klinische Entität, die sich durch *(1)* exklusive IgM‐Autoantikörper gegen die BMZ bei der direkten IF‐Mikroskopie, *(2)* serologische IgM‐Reaktivität gegen die epidermale Seite NaCl‐separierter humaner Spalthaut, *(3)* BP180 als Hauptzielantigen, *(4)* überwiegend nichtbullösen Phänotyp ohne Schleimhautbeteiligung sowie *(5)* insgesamt milden Krankheitsverlauf auszeichnet.

## DANKSAGUNG

Wir danken Yvonne Frambach (Lübeck, Deutschland) und Thomas Neumann (Hannoversch Münden, Deutschland) herzlich für die Bereitstellung klinischer Daten ihrer Patienten. Unser besonderer Dank gilt Ingeborg Atefi und Vanessa Krull, Lübeck, Deutschland, für ihre herausragende technische Unterstützung. Wir danken Christian Probst und Lars Komorowski von Euroimmun, Lübeck, Deutschland, für die Bereitstellung rekombinanter BP180‐Proteine.

Open access Veröffentlichung ermöglicht und organisiert durch Projekt DEAL.

## FINANZIERUNG

Diese Arbeit erhielt infrastrukturelle Unterstützung durch die DFG im Rahmen des Sonderforschungsbereichs SFB 1526 Pathomechanismen Antikörper‐vermittelter Autoimmunerkrankungen (PANTAU), Projektnummer 454193335, des schleswig‐holsteinischen Exzellenzclusters Präzisionsmedizin für chronische Entzündungserkrankungen (DFG EXC 2167/1) und Mittel der Universität zu Lübeck (CS06‐2019 an CMH).

## INTERESSENKONFLIKT

E.S. hat eine wissenschaftliche Kooperation sowie Patente mit Euroimmun, Lübeck, Deutschland. S.G., C.M.H. und N.v.B. halten ein Patent bei Euroimmun, Lübeck, Deutschland. Alle übrigen Autoren erklären, dass keine potenziellen Interessenkonflikte bestehen, einschließlich spezifischer finanzieller Interessen, Beziehungen oder Zugehörigkeiten, die im Zusammenhang mit dem Thema dieses Manuskripts relevant sind.

## Supporting information



Supplementary information
