# Supplementary material for: Klinisches und immunpathologisches Spektrum des Immunglobulin‐M‐Pemphigoids: eine multizentrische Fallserie
Source: J Dtsch Dermatol Ges. 2025 Dec 11;23(12):1555–65. [Article in German] doi: 10.1111/ddg.15838_g (PMC12697330; doi:10.1111/ddg.15838_g)
Supplement: Supplementary file 1 — Supplementary information [file DDG-23-1555-s001.docx]

**Ergänzendes Material (Supplementary Material)**

TABELLE S1 Detaillierte klinische Merkmale der Patienten mit IgM-Pemphigoid.

| **Fall #** | **Alter** | **Geschlecht** | **Zeitspanne bis zur Diagnose** | **Initiale Verdachtsdiagnose** | **Klinisches Bild** | | | | | | | | **Therapie** | | **Nachsorge (Monate)** | **Ergebnis** |
| --- | --- | --- | --- | --- | --- | --- | --- | --- | --- | --- | --- | --- | --- | --- | --- | --- |
|  |  |  |  |  | ***Erythem/ urtikarielle Läsionen*** | ***Erosionen*** | ***Blasen*** | ***Exkoriierte Papeln/ Plaques*** | ***Lichenifikation*** | ***Juckreiz*** | ***Hyperpigmentierung*** | ***Schleimhautbeteiligung*** | ***topische CS*** | ***systemisch*** |  |  |
| 1 | 98 | M | Mehrere Monate | Chronische Prurigo | + | + | – | + | + | + | – | – | + | - | 4 | PRMT |
| 2 | 92 | W | 9 Monate | Ekzem | + | + | +* | + | + | + | – | – | + | - | 13 | CROT |
| 3 | 75 | W | 26 Monate | Chronische Prurigo | + | – | – | + | – | + | – | – | + | Dapson | 28 | PRMT |
| 4 | 79 | M | 13 Monate | Bullöses Pemphigoid | + | + | – | + | – | + | – | – | + | - | 17 | PRMT |
| 5 | 60 | M | 4 Monate | Chronische Prurigo | + | – | – | + | + | + | + | – | + | *Initial*: Doxycyclin, Azathioprin  *Aktuell*: MMF, Prednisolon, Rituximab | 10 | CDA |
| 6 | 69 | W | 26 Monate | Chronische noduläre Prurigo | + | – | – | + | – | + | – | – | + | Prednisolon | n.d. | LTF |
| 7 | 70 | M | 2 Monate | HCT-assoziiertes AE | + | – | +* | – | – | + | – | – | + | - | 5 | CROT |
| 8 | 84 | M | 12 Monate | Parapsoriasis | + | – | – | – | + | + | – | – | + | Azathioprin^#^ | 8 | PRMT |
| 9 | 73 | M | 13 Monate | Chronische Prurigo | – | – | – | + | + | + | – | – | + | - | 10 | CROT |
| 10 | 84 | W | 12 Monate | Lamotrigin-assoziiertes AE | + | – | – | – | + | + | – | – | + | - | n.d. | LTF |

*Abk.:* AE, Arzneimittelexanthem; CDA (*control of disease activity*), Kontrolle der Krankheitsaktivität; CROT (*complete remission off therapy*), Komplettremission ohne Therapie; CS, Kortikosteroide; HCT, Hydrochlorothiazid; M, männlich; LTF (*lost to follow-up*), Verloren für Nachsorge; MMF, Mycophenolatmofetil; N.d., nicht durchgeführt; PRMT (*partial remission on minimal therapy*), Teilremission unter minimaler Therapie; W, weiblich.

*Anamnestisch: Keine Blasen wurden bei der Erstvorstellung oder bei den Nachuntersuchungen festgestellt; ^#^wurde zur Behandlung einer nicht-dermatologischen Erkrankung initiiert, verbesserte jedoch auch die Hautkrankheit.

TABELLE S2 Ausführliche histologische und immunpathologische Befunde der Patienten mit IgM-Pemphigoid.

| **Fall #** | **Direkte IF BMZ** | **Indirekte IF auf NaCl-separierter humaner Spalthaut** | **Immunoblot** | | | **Biochip** | | | **Histopathologische Merkmale** | | | | | | | | | |
| --- | --- | --- | --- | --- | --- | --- | --- | --- | --- | --- | --- | --- | --- | --- | --- | --- | --- | --- |
|  |  |  | ***BP180 NC16A IgM*** | ***BP180ec IgM*** | ***BP180(ec)3 (C-terminal) IgM*** | ***Indirekte IF auf Spalthaut*** | ***BP180 NC16A IgM*** | ***BP230 IgM*** | ***Parakeratose*** | ***Hyper-granulose*** | ***Hypogranulose*** | ***Akanthose*** | ***Spongiose*** | ***Neutrophile*** | ***Eosinophile*** | ***Lymphozytäre Infiltrate*** | ***Subepidermale Spaltbildung*** | ***Dermales Ödem*** |
| 1 | IgM (kein Muster) | Negativ | + | – | – | Negativ | – | – | + | – | – | + | – | + | – | + | + | – |
| 2 | IgM (kein Muster) | IgM Blasendach | – | – | – | IgM Blasendach | – | – | + | + | – | – | + | – | + | + | – | + |
| 3 | IgM (kein Muster) | IgM Blasendach | + | – | – | IgM Blasendach | + | – | – | – | – | – | – | – | + | + | – | – |
| 4 | IgM (kein Muster) | IgM Blasendach | – | – | – | IgM Blasendach | – | – | – | – | – | + | + | + | + | – | – | – |
| 5 | IgM (kein Muster) | IgM Blasendach | – | – | – | IgM Blasendach | – | – | – | – | – | + | – | – | – | + | – | – |
| 6 | IgM (kein Muster) | IgM Blasendach | + | + | – | Negativ | – | – | + | + | – | + | – | – | – | + | – | – |
| 7 | IgM (kein Muster) | IgM Blasendach | – | + | – | IgM Blasendach | – | – | – | – | – | – | + | – | + | + | – | + |
| 8 | IgM (kein Muster) | IgM Blasendach | – | – | – | Negativ | – | – | + | – | – | – | + | – | – | + | – | – |
| 9 | IgM  (n-Muster) | IgM Blasendach | – | – | – | IgM Blasendach | – | – | – | – | – | – | – | – | + | + | – | + |
| 10 | IgM (kein Muster) | IgM Blasendach | – | – | – | IgM Blasendach | – | – | + | – | + | + | + | + | + | + | – | – |

*Abk.:* BMZ, Basalmembranzone; ec, Ektodomäne; IB, Immunoblot; IF, Immunfluoreszenz

TABELLE S3 Analyse der IgM-Reaktivität mittels direkter IF-Mikroskopie der konsekutiven Proben von Patienten > 70 Jahre mit Verdacht auf eine BAID, jedoch ohne lineare Ablagerungen von IgG, IgA und C3 an der kutanen BMZ. Die Biopsien stammen aus potenziell sonnenexponierten Arealen.

| **Fall #** | **Alter** | **Geschlecht** | **IgM-Ablagerungen bei direkter IF-Mikroskopie** | | |
| --- | --- | --- | --- | --- | --- |
|  |  |  | *Arm* | *Bein* | *Stamm* |
| 1 | 82 | W |  |  | Neg. |
| 2 | 80 | W |  |  | Neg. |
| 3 | 76 | W |  | Neg. |  |
| 4 | 85 | M |  | Neg. |  |
| 5 | 87 | W |  | Neg. |  |
| 6 | 89 | W |  |  | Neg. |
| 7 | 77 | W |  |  | Neg. |
| 8 | 86 | W |  |  | Neg. |
| 9 | 85 | M | Neg. |  |  |
| 10 | 85 | M |  | Neg. |  |
| 11 | 84 | M | Neg. |  |  |
| 12 | 81 | W |  |  | Neg. |
| 13 | 90 | M |  |  | Neg. |
| 14 | 78 | W |  |  | Neg. |
| 15 | 81 | M |  |  | Neg. |
| 16 | 76 | W |  | Neg. |  |
| 17 | 84 | M | Neg. |  |  |
| 18 | 86 | M |  |  | Neg. |
| 19 | 86 | W |  |  | Neg. |
| 20 | 85 | M |  | Neg. |  |
| 21 | 82 | W |  | Neg. |  |
| 22 | 83 | M |  | Neg. |  |
| 23 | 79 | M |  |  | Neg. |
| 24 | 83 | W |  | Neg. |  |
| 25 | 75 | W | Neg. |  |  |
| 26 | 86 | M |  | Neg. |  |
| 27 | 88 | M |  | Neg. |  |
| 28 | 91 | M |  |  | Cyt. bod. |
| 29 | 88 | W |  |  | Neg. |
| 30 | 75 | W |  |  | Neg. |
| 31 | 88 | W |  | Neg. |  |
| 32 | 89 | W |  | Neg. |  |
| 33 | 79 | M |  |  | Neg. |
| 34 | 87 | W |  |  | Neg. |
| 35 | 84 | W | Neg. |  |  |
| 36 | 83 | M |  |  | Neg. |
| 37 | 86 | W |  | Neg. |  |
| 38 | 81 | W | Neg. |  |  |
| 39 | 84 | W |  | Neg. |  |
| 40 | 86 | W |  | Neg. |  |
| 41 | 83 | M |  |  | Neg. |
| 42 | 83 | W |  | Neg. |  |
| 43 | 90 | M |  | Neg. |  |
| 44 | 80 | M |  |  | Neg. |
| 45 | 88 | M |  | Neg. |  |
| 46 | 84 | W |  | Neg. |  |
| 47 | 85 | W | Neg. |  |  |
| 48 | 81 | W |  | Neg. |  |
| 49 | 86 | W |  | Neg. |  |
| 50 | 86 | W | Gran. |  |  |
| 51 | 80 | M | Neg. |  |  |
| 52 | 76 | W | Neg. |  |  |
| 53 | 85 | W |  | Neg. |  |
| 54 | 78 | M |  | Neg. |  |
| 55 | 91 | M |  |  | Neg. |
| 56 | 77 | W |  | Neg. |  |
| 57 | 91 | M |  | Neg. |  |
| 58 | 88 | W |  | Neg. |  |
| 59 | 87 | W |  |  | Neg. |
| 60 | 82 | W |  |  | Neg. |
| 61 | 93 | W | Neg. |  |  |
| 62 | 81 | W | Neg. |  |  |
| 63 | 89 | W | Neg. |  |  |
| 64 | 75 | M |  | Neg. |  |
| 65 | 87 | W | Neg. |  |  |
| 66 | 76 | W | Neg. |  |  |
| 67 | 86 | M |  | Neg. |  |
| 68 | 80 | W | Neg. |  |  |
| 69 | 76 | M |  |  | Neg. |
| 70 | 85 | W |  | Neg. |  |
| 71 | 86 | W |  |  | Neg. |
| 72 | 79 | M |  | Neg. |  |
| 73 | 80 | W |  | Cyt. bod. |  |
| 74 | 88 | M |  |  | Neg. |
| 75 | 80 | M |  | Cyt. bod. |  |
| 76 | 84 | M |  | Neg. |  |
| 77 | 78 | M | Neg. |  |  |
| 78 | 82 | W |  | Neg. |  |
| 79 | 87 | W |  | Neg. |  |
| 80 | 90 | W | Neg. |  |  |
| 81 | 94 | M |  |  | Neg. |
| 82 | 85 | M |  | Neg. |  |
| 83 | 79 | M |  |  | Neg. |
| 84 | 85 | M | Neg. |  |  |
| 85 | 81 | M |  | Neg. |  |
| 86 | 83 | M |  | Neg. |  |
| 87 | 84 | M |  | Neg. |  |
| 88 | 79 | M |  |  | Neg. |
| 89 | 82 | W |  |  | Neg. |
| 90 | 85 | M |  | Neg. |  |
| 91 | 82 | M |  |  | Neg. |
| 92 | 88 | M |  | Neg. |  |
| 93 | 78 | W |  | Neg. |  |
| 94 | 98 | M |  | Neg. |  |
| 95 | 86 | M |  |  | Neg. |
| 96 | 86 | W |  | Neg. |  |
| 97 | 83 | W |  |  | Neg. |
| 98 | 75 | M |  | Neg. |  |
| 99 | 86 | M |  |  | Neg. |
| 100 | 86 | W |  | Neg. |  |

*Abk.:* Cyt. bod., *cytoid bodies*; Gran., granulär; IF, Immunfluoreszenz; M, männlich; Neg., negativ; W, weiblich

TABELLE S4 Immunserologische Befunde bei Patienten von > 70 Jahren mit juckenden Dermatosen außer BAID.

| **Fall #** | **Alter** | **Geschlecht** | **Diagnose** | **IFSI Gruppe** | **Alter bei Diagnose** | **Pruritusintensität** | | **Indirekte IF auf Spalthaut (IgM)** | **Immunoblot mit BP180 NC16A (IgM)** |
| --- | --- | --- | --- | --- | --- | --- | --- | --- | --- |
|  |  |  |  |  |  | ***WI-NRS/ 24 h*** | ***AI-NRS/ 24 h*** |  |  |
| 1 | 83 | W | Arzneimittelexanthem | 1 | 77 | 10 | 8 | – | – |
| 2 | 81 | M | Atopische Dermatitis | 1 | 78 | 7 | 5 | – | – |
| 3 | 85 | M | Chronisch noduläre Prurigo (AD) | 3 | 84 | 10 | 8 | – | – |
| 4 | 85 | M | Chronischer Pruritus (AD) | 1 | 75 | 4 | 3 | – | – |
| 5 | 74 | W | Chronische Prurigo | 3 | 72 | 9 | 6 | – | – |
| 6 | 90 | W | Chronische Prurigo | 3 | 60 | 7 | 6 | – | – |
| 7 | 74 | W | Atopische Dermatitis | 1 | 70 | 9 | 8 | – | – |
| 8 | 74 | W | Chronische Prurigo | 3 | 70 | 8 | 5 | – | – |
| 9 | 71 | M | Chronische Prurigo | 3 | 70 | 10 | 8 | – | – |
| 10 | 77 | M | Atopische Dermatitis | 1 | 1 | 10 | 9 | – | – |
| 11 | 87 | W | Chronische Prurigo | 3 | 62 | 8 | 2 | – | – |
| 12 | 83 | M | Chronische Prurigo | 3 | 80 | 3 | 2 | – | – |
| 13 | 75 | W | Nummuläres Ekzem | 1 | 73 | 8 | 7 | – | – |
| 14 | 78 | M | Chronische Prurigo | 3 | 72 | 7 | 2 | – | – |
| 15 | 73 | M | Morbus Grover | 1 | 72 | 7 | 5 | – | – |
| 16 | 74 | M | Atopische Dermatitis | 1 | 70 | 6 | 3 | – | + |
| 17 | 73 | W | Lichen planus | 1 | 72 | 8 | 6 | – | + |
| 18 | 85 | M | Chronische Prurigo | 3 | 83 | 10 | 10 | – | – |
| 19 | 92 | M | Atopische Dermatitis | 1 | 91 | 5 | 3 | – | – |
| 20 | 81 | W | Chronische Prurigo | 3 | 67 | 10 | 10 | – | – |
| 21 | 80 | W | Atopische Dermatitis | 1 | 70 | 5 | 3 | – | – |
| 22 | 74 | W | Prurigo simplex | 1 | 74 | 7 | 5 | – | – |
| 23 | 83 | M | Asteatotisches Ekzem | 1 | 78 | 10 | 8 | – | – |
| 24 | 72 | W | Chronische Prurigo | 3 | 71 | 9 | 7 | – | – |
| 25 | 72 | M | Psoriasis | 1 | 50 | 8 | 7 | – | – |
| 26 | 82 | W | Atopische Dermatitis | 1 | 80 | 10 | 5 | – | – |
| 27 | 84 | M | Arzneimittelexanthem | 1 | 82 | 8 | 7 | + | – |
| 28 | 83 | W | Chronische Prurigo | 3 | 56 | 7 | 5 | – | – |
| 29 | 79 | W | Chronische Prurigo | 3 | 46 | 8 | 5 | – | – |
| 30 | 82 | M | Atopische Dermatitis | 1 | 77 | 6 | 5 | – | – |

*Abk.:* AD, atopische Dermatitis; AI-NRS, *Average Itch Numeric Rating Scale* (0, kein Juckreiz; 10, schlimmster vorstellbarer Juckreiz); BAID,, blasenbildende Autoimmundermatosen; IF, Immunfluoreszenz; IFSI, *International Forum for the Study of Itch* (Gruppe 1: Chronischer Pruritus auf primär läsionaler Haut; Gruppe 2: chronischer Pruritus auf primär nicht-läsionaler (unveränderter) Haut; Gruppe 3: chronischer Pruritus mit Kratzläsionen); M, männlich; W, weiblich; WI-NRS, *Worst Itch Numeric Rating Scale* (0, kein Juckreiz; 10, schlimmster vorstellbarer Juckreiz).

TABELLE S5 Immunserologische Befunde bei Patienten im Alter von > 70 Jahren mit Verdacht auf BAID, jedoch ohne lineare IgG-, IgA-, IgM- sowie C3-Ablagerungen an der kutanen BMZ.

| **Fall #** | **Indirekte IF auf humaner Spalthaut (IgM)** | **Immunoblot mit BP180 NC16A (IgM)** |
| --- | --- | --- |
| 1 | Neg. | Neg. |
| 2 | Neg. | Neg. |
| 3 | Neg. | Neg. |
| 4 | Neg. | Neg. |
| 5 | Neg. | Neg. |
| 6 | Neg. | Neg. |
| 7 | Neg. | Neg. |
| 8 | Neg. | Neg. |
| 9 | Neg. | Neg. |
| 10 | Neg. | Neg. |
| 11 | Neg. | Neg. |
| 12 | Neg. | Pos. |
| 13 | Neg. | Neg. |
| 14 | Neg. | Neg. |
| 15 | Neg. | Neg. |
| 16 | Neg. | Neg. |
| 17 | Neg. | Neg. |
| 18 | Neg. | Neg. |
| 19 | Neg. | Neg. |
| 20 | Neg. | Neg. |
| 21 | Neg. | Neg. |
| 22 | Neg. | Neg. |
| 23 | Neg. | Neg. |
| 24 | Neg. | Pos. |
| 25 | Neg. | Neg. |
| 26 | Neg. | Neg. |
| 27 | Neg. | Neg. |
| 28 | Neg. | Neg. |
| 29 | Neg. | Neg. |
| 30 | Neg. | Neg. |
| 31 | Neg. | N.d. |
| 32 | Neg. | N.d. |
| 33 | Pos. | Neg. |
| 34 | Neg. | N.d. |
| 35 | Neg. | N.d. |
| 36 | Pos. | Neg. |
| 37 | Neg. | N.d. |
| 38 | Neg. | N.d. |
| 39 | Neg. | N.d. |
| 40 | Neg. | N.d. |
| 41 | Neg. | N.d. |
| 42 | Neg. | N.d. |
| 43 | Neg. | N.d. |
| 44 | Neg. | N.d. |
| 45 | Neg. | N.d. |
| 46 | Neg. | N.d. |
| 47 | Neg. | N.d. |
| 48 | Neg. | N.d. |
| 49 | Neg. | N.d. |
| 50 | Pos. | Neg. |
| 51 | Neg. | N.d. |
| 52 | Neg. | N.d. |
| 53 | Neg. | N.d. |
| 54 | Neg. | N.d. |
| 55 | Neg. | N.d. |
| 56 | Neg. | N.d. |
| 57 | Neg. | N.d. |
| 58 | Neg. | N.d. |
| 59 | Neg. | N.d. |
| 60 | Neg. | N.d. |

*Abk.:* IF, Immunfluoreszenz; N.d., nicht durchgeführt; Neg., negativ; Pos., positiv
